# Translational and Rotational Postural Aberrations Are Related to Pulmonary Functions and Skill-Related Physical Fitness Components in Collegiate Athletes

**DOI:** 10.3390/jcm12144618

**Published:** 2023-07-11

**Authors:** May Tamim, Ibrahim M. Moustafa, Gopala K. Alaparthi, Paul A. Oakley, Deed E. Harrison

**Affiliations:** 1 Department of Physiotherapy, College of Health Sciences, University of Sharjah, Sharjah 27272, United Arab Emirates; mtamim@sharjah.ac.ae (M.T.); iabuamr@sharjah.ac.ae (I.M.M.); galaparthi@sharjah.ac.ae (G.K.A.); 2 Neuromusculoskeletal Rehabilitation Research Group, RIMHS—Research Institute of Medical and Health Sciences, University of Sharjah, Sharjah 27272, United Arab Emirates; 3 Senior Lecturer, Department of Health Professions, Manchester Metropolitan University, Manchester M15 6BH, UK; 4 Kinesiology and Health Sciences, York University, Toronto, ON M3J 1P3, Canada; docoakley.icc@gmail.com; 5 Independent Researcher, Newmarket, ON L3Y 8Y8, Canada; 6 CBP Nonprofit (A Spine Research Foundation), Eagle, ID 83616, USA

**Keywords:** posture, athletic performance, athletes, cardiopulmonary performance

## Abstract

This study assessed the relationship between body posture displacements, cardiopulmonary exercise testing (CPET), and skill-related physical fitness tests. One hundred male (60%) and female collegiate athletes (22.2 ± 4 yrs) with normal body mass indexes (BMI up to 24.9) were assessed via the PostureScreen Mobile^®^ app to quantify postural displacements such as head, thorax, and pelvis rotations and translations. CPET and physical performance tests, including the agility *t*-test, vertical jump test, stork static balance test (SSBT), and dynamic Y-balance test (YBT), were performed. Spearman correlation (r) and *p*-values are reported. The postural parameters were found to have moderate-to-high associations with the CPET and agility test, moderate correlations with the vertical jump test and SSBT (head and pelvic postures only), and weak correlations with the YBT. As the postural parameters were more asymmetric, both the CPET and performance skills scores were worse. For example: (1) a medium positive correlation was found between cranio-vertebral angle (CVA) and the vertical jump test (r = 0.54; *p*-value < 0.001) and SSBT (r = 0.57; *p*-value < 0.001), while a strong negative correlation was found between CVA and the agility test (r = −0.86; *p*-value < 0.001). (2) A strong positive correlation was found between CVA and oxygen uptake efficiency slope, load watts VO2 at VT, VO2/kg, and load watts at the respiratory compensation point (RCP) (r = 0.65 and r = 0.71; *p* < 0.001). Conversely, a significant negative correlation was found between CVA and VE/VO2 at VT (r = −0.61; *p* < 0.001). Postural rotations and translations of the head, thorax, and pelvis were statistically correlated with the physical performance skills and CPET in the young collegiate athletes. There were moderate-to-high associations with cardiopulmonary functions and the agility tests, moderate correlations with the vertical jump test, and weak correlations with the YBT. Postural alignment may be important for optimal physical performance and optimal cardiopulmonary function. Further research is necessary to elucidate the reasons for these correlations found in our sample of young and healthy athletes.

## 1. Introduction

Athletic performance is influenced by various factors such as strength, endurance, and skill [1,2,3]. However, postural alignment is often overlooked, despite being a critical component in optimal performance [4,5]. Proper posture is essential for athletes to perform at their best and minimize the risk of injuries. Unfortunately, many athletes develop postural aberrations due to various reasons, including repetitive movements and the overuse of certain muscle groups [5,6,7,8,9]. While many studies have suggested an association between postural abnormalities and exposure to athletic training across various age groups and different sports, the data available in this area are mostly limited to sagittal profiles [6,7,8]. For instance, several studies have shown an association between increased thoracic kyphosis, lumbar lordosis angles, and exposure to athletic training [5,9].

Postural aberrations can have a negative impact on both respiratory function and physical fitness components, which, in turn, can lead to decreased athletic performance and an increased risk of injury [10,11,12]. Few studies have examined the relationship between posture and pulmonary function or physical fitness [12,13]. A recent study by Moustafa et al. [13] reported that college athletes with forward head posture exhibited less efficient physical fitness and altered sensorimotor processing and integration compared to athletes with a normal sagittal head posture alignment [13]. Another study showed a correlation between sagittal pelvic balance and the incidence of acute and micro-traumatic injuries of the pelvic-femoral complex [14]. There are also studies that have shown that sagittal spinal subluxations can lead to decreased pulmonary function, suggesting a direct relationship between spinal subluxation and pulmonary function through a somatovisceral reflex pathway via the sympathetic nervous system [15,16].

In 1980, Harrison detailed the possible postural displacements of the head, thorax, and pelvis as rotations and translations with 12 simple motions (6 possible rotations and 6 translations in each region) in 6 degrees of freedom [17,18]. This description of postural displacements as rotations and translations allows for a precise analysis of how posture influences spine tissues, spine kinematics, and injury mechanisms. However, still today, this comprehensive analysis of posture is seldom applied in studies due to the complexities of its measurements and likely the tediousness of assessing and comparing the multiple variables. For example, most often, investigations on postural relevance limit their analysis to one plane (sagittal, coronal, or transverse) or one region (head, thorax, or pelvis) and do not fully assess all translational and rotational posture displacements [17,18,19]. To the best of our knowledge, there are currently no studies that have investigated this relationship within the context of translational and rotational posture aberrations, specifically among collegiate athletes. These were considered biomechanically, as the movements of the spine are intricate and involve complex coupling patterns that are influenced by the biomechanical characteristics between two segments [20,21]. A translation or rotation of the spine in one geometric plane can lead to simultaneous movements in other planes [22,23,24]. This emphasizes the importance of conducting a global posture assessment that considers translational and rotational displacements, as suggested by Harrison et al. [19]. As postural aberrations can occur in all planes, not just the sagittal plane, there is a need for a more comprehensive understanding of the relationship between 3D posture parameters and pulmonary function to enhance athlete development and training.

Recent technological advancements have made it possible to accurately measure upright posture as translational and rotational displacements of the head, thoracic cage, and pelvis [25,26]. These advancements present a unique opportunity to identify the potential areas of postural abnormalities for interventions that can improve pulmonary function and athletic performance. Thus, the aim of this study is to provide a more comprehensive understanding of the relationship between 3D posture parameters, pulmonary function, and athletic performance, which may be essential to improving athlete development and training. Our hypothesis is that multiple (more than one plane and more than one region) postural displacements of the head, ribcage, and pelvis will impact and alter a specific set of athletic skills and cardiopulmonary measures. Our objective is to identify which (if any) of these postural rotations and translations have the potential to influence these measures, such that future interventional trials might consider these findings as outcome assessments.

## 2. Materials and Methods

One hundred healthy male and female collegiate athletes were recruited in this study. The inclusion criteria were: (i) ages between 17 to 26, and (ii) a normal body mass index (BMI) of up to 24.9. Ethical approval was obtained from the Ethics Committee of the University (REC-22-11-26-S) and informed consent was obtained from all the participants prior to the data collection, in accordance with the relevant guidelines and regulations. All the participants were screened for the following exclusion criteria: (i) inflammatory joint disease or other systemic pathologies; (ii) a prior history of overt injury and surgery relating to the musculoskeletal system or a disorder related to the spine and extremities; and (iii) musculoskeletal pain in the previous three months.

### 2.1. Outcome Measures

#### 2.1.1. Posture Measurement

**Figure 1:**
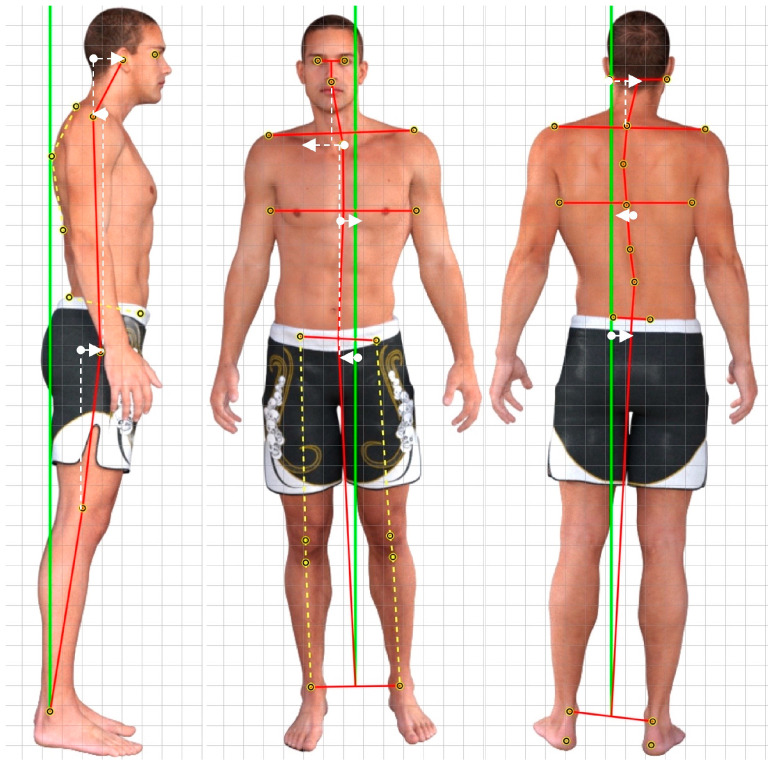
PostureScreen^®^ Mobile app (PSM) measurements of the sagittal plane and coronal plane posture displacements for the head, thoracic cage, and pelvis. Depicted is the general method of measurement for translations of the head relative to the thorax, the thorax relative to the pelvis, and the pelvis relative to the knees and feet. The green line represents a true vertical plumbline alignment, the red lines indicate the body segment displacements, and the white arrows indicate the direction of translation of each region.

The postural examinations were carried out with a photographic method using the PostureScreen^®^ Mobile app (PSM). The PSM application has been shown to be a reliable and valid method for evaluating static posture [25,26]. PSM is a mobile application designed to measure the posture parameters in individuals. The app captures images of the participant from various angles, including anterior and posterior (coronal plane) and left and right (sagittal plane). The app calculates posture variables by digitally marking an individual’s anatomical points, which may vary depending on the number of variables of interest. The process of digitally marking these points consists of identifying and demarcating anatomical reference points, such as the pelvic iliac spines, greater trochanter, femoral condyle, and tragus, directly on the mobile device screen. The app then calculates the body angles and distances based on these marked points. The output file provided by the app includes values of the posture variables and images that illustrate the marked points and their locations in relation to a neutral posture. These output files can be used to compare and analyze the posture variables among the participants. Figure 1 depicts the sagittal plane and coronal plane measurements of the head, thoracic cage, and pelvis rotations and translations used in this study.

#### 2.1.2. Cardiopulmonary Exercise Testing (CPET)

**Figure 2:**
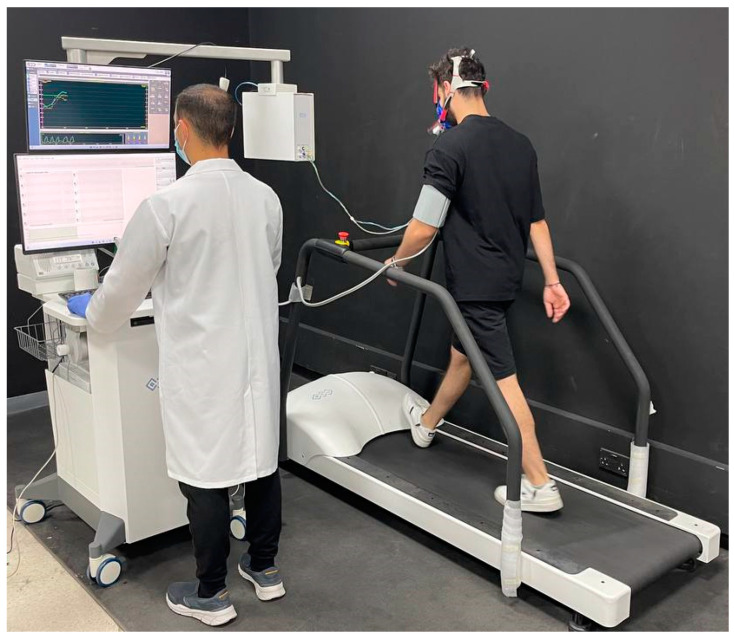
Cardiopulmonary exercise testing setting.

Cardiopulmonary exercise testing (CPET) performance analyses were conducted within the recommendations of the American Heart Association (AHA) [27,28]. All the CPETs were carried out in a single laboratory, where the participants underwent an intensity-graded, maximal effort exercise test with continuous gas exchange in the absence of chest pain and ECG abnormalities (Figure 2). The device used was calibrated before each test. A treadmill was the method used in the tests, taking into consideration the population of the study and correlating the results with a possible involvement of spinal malalignment [29,30,31]. The treadmill and surrounding equipment were thoroughly disinfected after every test (including but not limited to the handlebars, hand holds, and rails). All the participants were instructed to use the emergency stop buttons located on both sides of the treadmill ergometer in the event where they were unable to finish the test for any given reason. In the event of a termination of the exercise test, an assessment of dyspnoea and leg effort was recorded using the modified Borg scale and the cause of the termination of the test was recorded [32].

All the performance data, including blood pressure, were recorded continuously during the tests. For the ventilatory parameters, we considered the VE/VCO2 slope (ventilatory efficiency), VO2 (mL/min) at VT (tidal volume or VT is the volume of gas inspired and expired during one respiratory cycle), VO2/kg (maximal oxygen uptake), and VE I/min (VE/VO2 at VT) (ventilatory equivalents for oxygen) [33]. All the ventilatory parameters were recorded through a respiratory valve and mouthpiece incorporating a gas analyzer.

A maximal CPET protocol was carried out on the treadmill (800 series, BTL Industries Cardio point, UK) with sport-specific incremental protocols. Calibration was carried out before the start of the tests and repeated as needed during the day. The calibration included checking for the environmental carbon dioxide (CO_2_) measurement, calibrating the gas analyzers, flow sensor zeroing, and flow calibration. We started the protocol with a single minute sitting/resting phase, automatically followed by a 2 min flat walk at 6 km/h as a warm-up setting. After the 2 min flat walk phase, the treadmill speed was increased automatically by continuous 8 and up to 10 km/h inclined running, with an increasing slope of 1.0% at every minute until participant exhaustion was recorded. This protocol was pre-set before the start of the test. After termination of the running (VT), measurements were continuously taken for 1 min at a 4 km/h walk, followed by a 4 min rest [32,34,35].

#### 2.1.3. Athletic Performances

Agility and speed, leg power, static balance, and dynamic balance were evaluated as indicators of athletic performance.

##### Agility: *T*-Test

**Figure 3:**
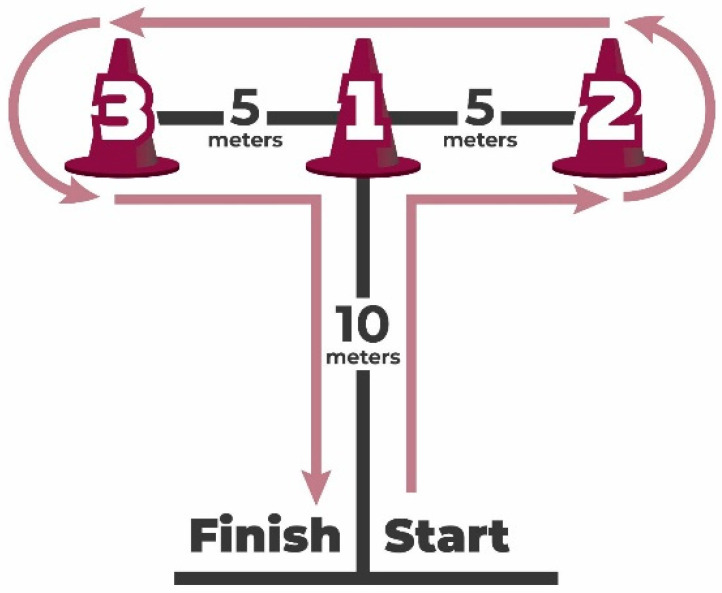
Agility *t*-test.

The agility *t*-test, a test of four-directional running speed that demands quick changes in direction while maintaining speed and balance, was used to assess agility. The validity of the T-test, in comparison to the 40-yard dash and hexagon test, has been well-established [36]. A statistical analysis was performed on the quickest of three trials. The trial times were tracked to the nearest tenth of a second (See Figure 3).

##### Leg Power: Non-Countermovement Vertical Jump Test

**Figure 4:**
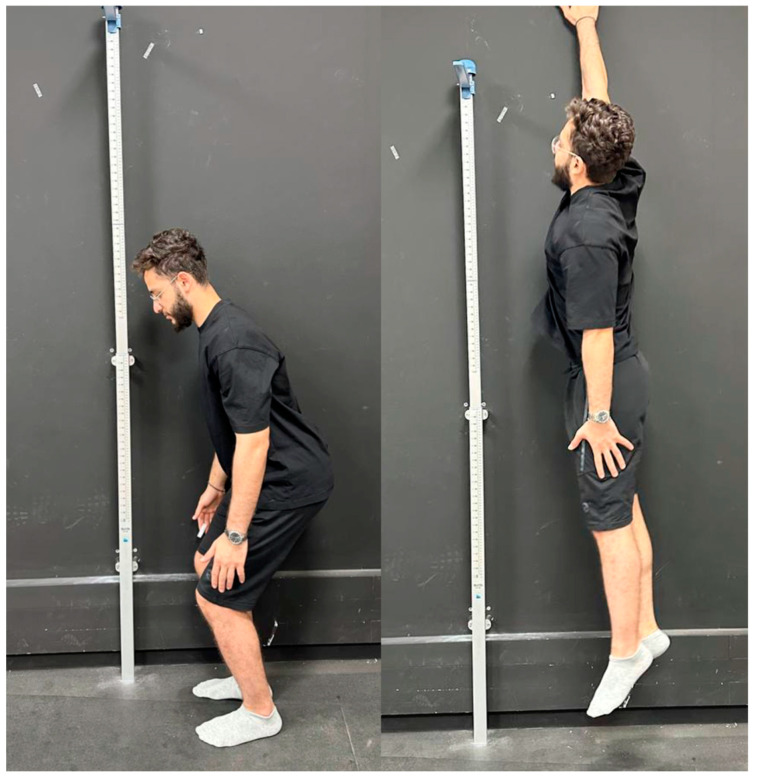
Leg power: non-countermovement vertical jump test.

The non-countermovement vertical jump test, often referred to as the squat jump or static jump test, was used to assess leg power [37]. The participants were positioned in a shoulder-width apart stance. They were requested to squat for a brief second with their knees flexed to 70°, then leap as high as they could. By placing one hand on the wall, the participants marked the height of their jump. The vertical jump height was determined to be the mean of the three trials (Shown in Figure 4).

Static Balance: Stork Static Balance Test

Static balance was evaluated using the stork static balance test [38]. With both hands on their hips and their opposite foot resting against their standing knee, the participants stood on their stronger leg and lifted the heel of their standing leg off the ground at the “go” signal. The participants were asked to maintain this position for as long as possible. The best result out of 3 trials was used. The test was terminated when the raised heel landed on the ground or when the opposing foot moved away from the knee. There is evidence that the test–retest reliability was in the clinically desirable range [38].

##### Dynamic Balance: Y-Balance Test or YBT

**Figure 5:**
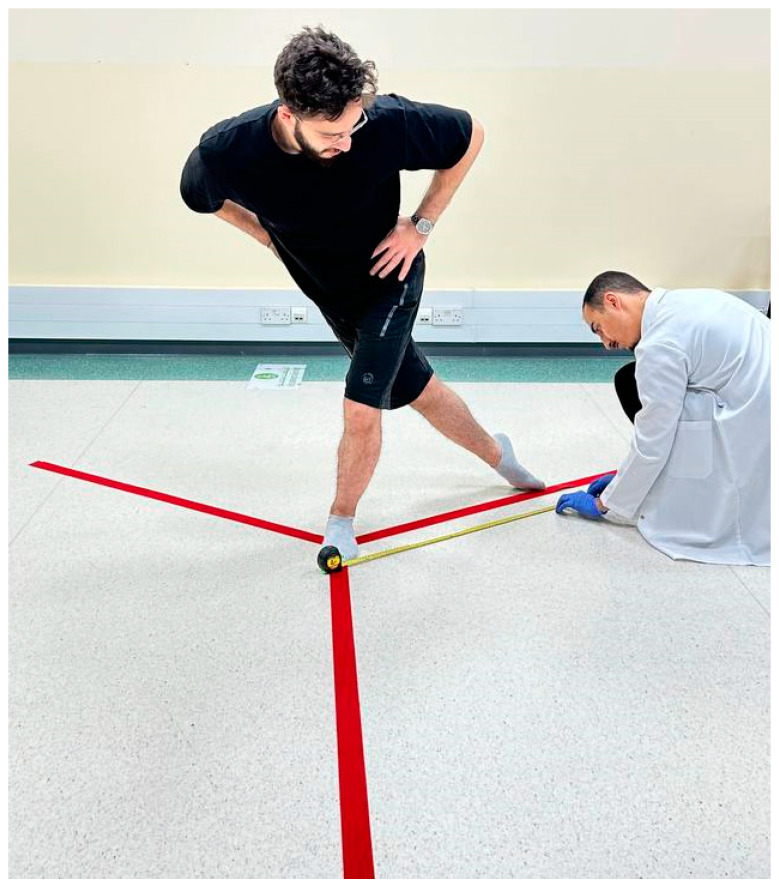
Y-balance test.

The participant’s dominant leg was evaluated for dynamic balance using the lower quarter Y-balance test (YBT) [38]. The participants’ leg lengths were first measured while lying supine, measuring from the anterior superior iliac spine to the most distal aspect of the medial malleolus. The participants were instructed to stand on their dominant leg with the great toe placed at the center of installed floor marking tapes aligned in 3 directions (anterior, postero-medial, and postero-lateral). The two posterior lines were extended at angles of 135° from the anterior line. The participants were asked to go in three directions while maintaining a single-limb stance (shown in Figure 5). The trials were repeated if (a) the reaching foot did not touch the required line while maintaining a single-limb stance, (b) the stance foot was lifted from the center, (c) their balance was lost at any point, (d) the participants did not maintain the start and return positions for one full second, and/or (e) they touched the reaching foot to gain support. Their maximal reach was measured in each direction. The composite score (CS) was calculated as: CS = ([maximum anterior reach distance + maximum postero-medial reach distance + maximum postero-lateral reach distance]/[leg length × 3] × 100).

$$\frac{\left(maximum\;anterior\;distance\;+\;maximum\;posteromedial\;distance\;+\;maximum\;posterolateral\;distance\right)}{3\times leg\;length}\times 100$$

Three trials were conducted in each direction with a rest interval for 2 min and the best out of these three trials was used. The test–retest reliability (ICC’s) for the different reach directions ranged between 0.90 and 0.95 [38].

### 2.2. Data Analysis

#### 2.2.1. Sample Size Determination

A statistical analysis was then performed using SPSS statistical software version 25. A Fisher Z transformation was utilized for a sample size estimation with the power set at 0.80, the beta set at 0.20, and the alpha set at 0.05. The estimated needed sample size for correlation was at least 74. A larger sample size of 100 was used to ensure external validity and strengthen the study.

#### 2.2.2. Statistical Analysis

Descriptive data are presented as mean ± standard deviation. To determine the normality of the collected numerical variables, the Shapiro–Wilk test was used; however, none of the numerical variables satisfied the parametric assumptions, so they are described using the median and interquartile range (IQR). Next, a spearman correlation was performed, and the correlation coefficient (r) and *p*-values are reported to demonstrate the relationship between the posture parameters and physical performance skills, as well as the posture parameters and cardiopulmonary functions. The correlation coefficient can be any number between −1 and 1, where a negative sign indicates a negative correlation and a positive sign indicates a positive correlation. In terms of the association, for absolute values of r, 0–0.19 was regarded as very weak, 0.2–0.39 as weak, 0.40–0.59 as moderate, 0.6–0.79 as strong, and 0.8–1 as a very strong correlation. The level of significance was set at 0.05 and the correlation was considered to be statistically significant when the *p*-value was less than 0.05.

## 3. Results

### 3.1. Participant Demographics and Characteristics

The participant characteristics are shown in Table 1. The Shapiro–Wilk test was used to test for the normality of the numerical variables and all the numerical variables did not follow the parametric assumptions, so they are described using median and interquartile range (Table 2, Table 3 and Table 4).

### 3.2. Correlations between Variables

#### 3.2.1. Correlations between Posture and Performance Skills

Several correlations were identified between the posture parameters and physical performance skills (Table 5 and Table 6). A medium positive correlation was found between cervical vertebral angle (CVA) and the vertical jump test (r = 0.54; *p*-value < 0.001), while a strong negative correlation was found between CVA and the agility test (r = −0.86; *p*-value < 0.001). A medium positive correlation was also identified between CVA and the stork balance test (r = 0.57, *p*-value = < 0.001). A negative medium correlation was found between the stork balance test and lateral head translation, as well as anterior head translation (AHT) (r = −0.46, −0.51, respectively, *p*-value = <0.001, <0.001, respectively). There was, however, a very weak correlation between the stork balance test and lateral angulation head, and a weak correlation with thoracic kyphosis (r = −0.19, r = −0.27, respectively, *p*-value = 0.13, 0.002, respectively).

There was also a negative medium correlation between lateral translation head sagittal and the y-balance test anterior left and y-balance test posterolateral left and right (r = −0.35, −0.42, −0.40, respectively, *p*-value = 0.004, <0.001, <0.001, respectively), and a strong positive correlation between lateral translation head sagittal and the agility test (r = 0.786; *p*-value < 0.001). A negative medium correlation was found between anterior head translation (AHT) coronal and the y-balance test anterior left, y-balance test posterolateral left and right, and y-balance posteromedial left and right (r = −0.44, −0.48, −0.46, −0.47, and −0.39, respectively, *p*-value < 0.001), and a strong positive correlation was found between AHT coronal and the agility test (r = 0.81; *p*-value < 0.001). Additionally, a strong positive correlation was found between lateral angulation head and the agility test (r = 0.74; *p*-value < 0.001), thoracic kyphosis and the agility test (r = 0.695; *p*-value < 0.001), and rib anterior translation coronal and the agility test (r = 0.75; *p*-value < 0.001).

Furthermore, a medium negative correlation was found between pelvic tilt and the vertical jump test (r = −0.54; *p*-value < 0.001), while a significant positive strong correlation was found between pelvic tilt and the agility test (r = 0.79; *p*-value < 0.001). There was, however, a weak negative correlation between pelvic tilt and the stork balance test (r = −0.35, *p*-value = 0.02). A negative medium correlation was found between hip pelvis lateral angulation and the vertical jump test, as well as with the stork balance test (r = −0.48, −0.44, respectively; *p*-value < 0.001), and a positive strong correlation was found between hip pelvis lateral angulation and the agility test (r = 0.79; *p*-value < 0.001). Similarly, there was a medium negative correlation between hip pelvis anterior translation coronal and the vertical jump test, as well as with the stork balance test (r = −0.65, −0.47, respectively; *p*-value < 0.001), and a positive strong correlation was found between hip pelvis anterior translation coronal and the agility test (r = 0.78; *p*-value < 0.001). Lastly, there was a medium negative correlation between hip pelvis lateral translation and the vertical jump test (r = −0.56; *p*-value < 0.001), while a significant positive strong correlation was found between hip pelvis lateral translation and the agility test (r = 0.73; *p*-value < 0.001).

#### 3.2.2. Correlations between Posture Parameters and Cardiopulmonary Measures

There were also significant correlations between the posture parameters and cardiopulmonary functional parameters identified (Table 7 and Table 8). A strong positive correlation was found between CVA and oxygen uptake efficiency slope, load watts VO2 at VT, VO2/kg, and load watts at the respiratory compensation point (RCP), (r = 0.65 and r = 0.71; *p* < 0.001). Conversely, a significant negative correlation was found between CVA and VE/VO2 at VT (r = −0.61; *p* < 0.001). Furthermore, there was also a strong negative correlation between lateral translation head sagittal and oxygen uptake efficiency slope, load watts VO2 at VT and VO2/kg, and load watts at RCP; (r = −0.62 and r = −0.72, *p* < 0.001, respectively), and a strong positive correlation between lateral head translation and VE/VO2 at VT (r = 0.65; *p* < 0.001).

## 4. Discussion

This investigation sought the association between postural displacements such as translations and rotations of the head, thorax, and pelvis in relation to both cardiopulmonary exercise tests and physical performance skills. Our hypothesis that multiple (more than one plane and more than one region) postural displacements would impact and alter a specific set of athletic skills and cardiopulmonary measures was supported by our primary findings. In our young collegiate athlete populations, we identified many statistically significant correlations between posture displacements of the head, thoracic cage, and pelvis and their cardiopulmonary and athletic skills, including moderate-to-high associations with cardiopulmonary function measures and agility testing, moderate correlations with the vertical jump test and stork balance test, and weak correlations with the YBT. The differences in both the cardiopulmonary and physical performance skills based on the postural parameters in this young and healthy population add to the growing body of literature showing the importance of posture; that is, a poor postural alignment has been associated with negative effects in asymptomatic populations [12,13]. Specifically, we identified the several key postures of the head (forward head, lateral translation, and lateral bending), thoracic spine (increased kyphosis and anterior translation), and pelvis (flexion/extension, anterior translation, lateral translation, and lateral bending) that have the strongest influence on athletic skills and cardiopulmonary exercise tests. Our findings indicated that it is oversimplified to assess posture in one region or one plane and that, at a minimum, multiple planes in a single region should be investigated in future investigations for a more complete analysis.

A recent study assessing sensorimotor integration and somatosensory processing variables between asymptomatic individuals with and without forward head posture found that the CVA was significantly correlated with all the measured neurophysiological variables, indicating that, as forward head posture increased, the sensorimotor integration and somatosensory evoked potential (SEP) processing became less efficient [39]. Regarding sport performance, two recent investigations have identified that postural profiles in the sagittal plane are associated with athletic skills and functional performance measures [12,13]. For instance, Moustafa and colleagues [13] found that college athletes with increased forward head posture exhibited both altered sensorimotor processing and integration measures and less efficient skill-related physical skills versus athletes without forward head posture. The previous investigation assessing sensorimotor integration and SEPs [39] provides a neurophysiological explanation of the results herein and suggests that posture is an important determinant of peak physical performance.

### 4.1. Posture and Athletic Skill Measures

The question arises of how asymmetry in posture, in terms of rotations and translations, contributes to decrements in physical performance skills? First, any asymmetric posture automatically changes the three-dimensional length-tension relationships of the muscles and tendons [40], and because peak athletic performance translates into differences in fractions of seconds and/or millimeters, poor postures could translate into small but significant decrements in athletic skill performance. Studies have shown that postural alignment affects muscle force production, as measured using electromyography (EMG). Kumar and Naraya [41], for example, determined that having a pre-rotated thoracic posture resulted in reduced torque generation. Further, Roy et al. [40] determined that an initial trunk flexion position determined the normalized L3 EMG activity and trunk extension torque indicative of neuromuscular efficiency; that is, trunk muscle synergism is modulated by posture.

Many studies have found differences between athletes and non-athletes in body posture [42,43], as well as differences in the postures between athletes competing in different sports [44,45,46]. It may be that habitual postures adopted during athletic performance lead to sport-specific permanent changes in postural alterations, as Kruusamäe et al. [45] suggested. This may be due to the postural muscle strengthening resulting from repeated intensive exercise [46]. An important consideration may be that, regardless of being an athlete, poor asymmetric body postures may be common, and that these asymmetric postures adversely affect body dynamics. Grabara [42], for example, found postural alterations in both athletes and controls. Certainly, the structure of the posture determines its dynamics [47], and the results from the current study support the notion of quite clear relationships between physical performance measures and body postures; that is, better postures are associated with better performances. Thus, a poor postural alignment creates a non-ergonomic disequilibrium about the gravity line [48] that, in turn, changes the muscle length-tension relationships and results in changes in physical performance.

### 4.2. Posture and Cardiopulmonary Exercise Tests

The cardiopulmonary exercise tests, including a greater VO2 at VT, greater VO2/kg, and smaller VE L/min, all demonstrated poorer values and were statistically significantly correlated to asymmetric postures for all the posture parameters assessed (reported in Table 7 and Table 8 and shown in Figure 1). In other words, the larger the postural displacements, the worse the cardiopulmonary measures; these were of moderate-to-high relationships, with most being r = 0.6–0.7, *p* < 0.001. Posture as assessed as translations and rotations of the head, thorax, and pelvis has not, to our knowledge, previously been correlated with cardiopulmonary functional measures, and it is not known how posture and cardiopulmonary measures are related. We speculate, however, that one of the mechanisms responsible for some of the main effects has to do with how posture alterations in the head and cervical spine influence respiratory mechanics [49,50]. For example, in a pilot project using spirometry, Kapreli and colleagues [49] identified a strong correlation between forward head posture (FHP) and reduced respiratory muscle strength in chronic neck pain patients. Changing the position of the head causes disturbances in the three-dimensional (3D) shape of the chest and its respiratory movements. Szczygiel et al. [50] utilized a 3D photogrammetric system and found that FHP caused limited movement of the lower ribcage during respiration in all three planes: sagittal, frontal, and transverse, while lateral tilt of the head caused participants to have a reduced lower chest expansion and increased amplitude of respiratory movements in the upper chest [50]. Still, future research is needed to determine the specific reasons for the changes in the cardiopulmonary measures identified in the current investigation and the implications of the complex relationship between altered posture and cardiopulmonary findings.

### 4.3. Limitations

There are several limitations of this current investigation, all pointing to possible future works. First, because we chose a young adult population comprising asymptomatic collegiate athletes, it is not possible to extrapolate our results to different decade of life populations and/or musculoskeletal pain populations. Second, this study did not address the causal relationship between abnormal postures, cardiopulmonary function, and skill-related physical fitness. Future investigations are needed to determine this causal relationship and refute or confirm our study’s proposed limitations and their possible relevancies. Furthermore, it is unknown if rehabilitation programs guided by both altered postural alignment and functional disturbances would theoretically benefit and perhaps have greater success in the treatment of spinal disorders and decreased athletic and cardiopulmonary performance measures.

## 5. Conclusions

Posture parameters, in terms of rotations and translations of the head, thorax, and pelvis, were statistically correlated with physical performance skills and cardiopulmonary function. There were moderate-to-high associations with the cardiopulmonary exercise tests and agility tests, moderate correlations with the vertical jump test, and weak correlations with the YBT. Specifically, we identified several key postures of the head (forward head, lateral translation, and lateral bending), thoracic spine (increased kyphosis and anterior translation), and pelvis (flexion/extension, anterior translation, lateral translation, and lateral bending) that have an influence on athletic skills and cardiopulmonary exercise tests. These specific postures should be considered in future interventional trials as outcome assessments that might influence human performance. Further research is necessary to elucidate the reasons for the correlations found in our sample of young and healthy athletes.

## Figures and Tables

**Table 1:** Descriptive data for the demographic variables are presented. The values are presented as mean and standard deviation (SD) for age and weight. BMI = body mass index. ^a^ The athletic activity in which the participant was involved.

Variable	FHP (*n* = 50)
Age (years)	22.2 ± 4
Weight (kg)	62.5 ± 3
BMI	18.2 ± 1.5
Gender (%)	
Male	60
Female	40
Sport ^a^, number in percent (%)	
Handball	20
Volleyball	15
Basketball	25
Football	30
Other	10
Smoking	
Non smoker	70
Smoker	30
Race/ethnicity	
Asian	64
African	36

**Table 2:** Descriptive statistics of posture parameters as the median and interquartile range (IQR). CVA = craniovertebral angle.

Posture Parameters	N = 100 Median (IQR)
CVA (°)	52.0 (49.0, 56.5)
Lateral translation head sagittal (cm)	2.00 (1.00, 3.80)
AHT coronal (cm)	0.90 (0.25, 1.07)
Lateral angulation head (°)	11.6 (10.0, 15.8)
Thoracic kyphosis (°)	22.0 (20.0, 30.0)
Rib anterior translation coronal (cm)	0.50 (0.00, 0.70)
Pelvic tilt (°)	9.80 (7.20, 12.00)
Pelvis lateral angulation (°)	2.90 (1.50, 4.90)
Pelvis anterior translation coronal (cm)	0.40 (0.10, 0.70)
Hip pelvis lateral translation (cm)	2.10 (1.00, 2.50)

**Table 3:** Descriptive statistics of physical performance skills reported as the median and interquartile range (IQR).

Physical Performance Skills	N = 100 Median (IQR)
Static stork test results	55 (45, 64)
Y-balance test (cm) anterior for the left side	94 (75, 109)
Y-balance test (cm) anterior for the right side	98 (74, 107)
Y-balance test (cm) posterolateral for the left side	100 (86, 112)
Y-balance test (cm) posterolateral for the right side	98 (89, 111)
Y-balance test (cm) posteromedial for the left side	95 (86, 109)
Y-balance test (cm) posteromedial for the right side	99 (82, 110)
Vertical jump test (cm)	48 (39, 55)
Agility test (s)	4.97 (9.60, 11.70)

**Table 4:** Descriptive statistics of cardiopulmonary functions reported as the median and interquartile range (IQR).

Cardiopulmonary Functions	N = 100 Median (IQR)
VE/VCO2 slope	34.2 (31.4, 39.0)
VO2 (mL/min) at VT	2809 (1788, 3112)
VO2/kg	34 (30, 44)
VE I/min (VE/VO2 at VT)	91 (87, 113)

**Table 5:** Spearman correlation coefficient (r) and *p*-values (*p*) between CVA, lateral translation head sagittal, AHT coronal, lateral angulation head, and thoracic kyphosis (first row), and each of the physical performance skills (first column).

Physical Performance Skills	CVA		Lateral Translation Head Sagittal		AHT Coronal		Lateral Angulation Head		Thoracic Kyphosis	
	r	*p*	r	*p*	r	*p*	r	*p*	r	*p*
Stork balance test (s)	0.57	<0.001	−0.46	<0.001	−0.51	<0.001	−0.19	0.13	−0.27	0.02
Y-balance test (cm) anterior for the left side	0.33	<0.001	−0.35	0.004	−0.44	<0.001	−0.22	0.075	−0.28	0.025
Y-balance test (cm) anterior for the right side	−0.09	0.418	0.17	0.166	0.02	0.854	0.21	0.085	0.12	0.348
Y-balance test (cm) posterolateral for the left side	0.39	0.001	−0.42	<0.001	−0.48	<0.001	−0.31	0.013	−0.28	0.024
Y-balance test (cm) posterolateral for the right side	0.36	0.003	−0.403	<0.001	−0.46	<0.001	−0.32	0.01	−0.27	0.027
Y-balance test (cm) posteromedial for the left side	0.33	0.007	−0.39	0.001	−0.47	<0.001	−0.27	0.028	−0.23	0.064
Y-balance test (cm) posteromedial for the right side	0.29	0.016	−0.29	0.015	−0.39	0.001	−0.19	0.13	−0.19	0.12
Vertical jump test (cm)	0.54	<0.001	−0.51	<0.001	−0.62	<0.001	−0.33	0.007	−0.32	0.008
Agility test (s)	−0.86	<0.001	0.786	<0.001	0.81	<0.001	0.74	<0.001	0.695	<0.001

**Table 6:** Spearman correlation coefficient (r) and *p*-values (*p*) between rib anterior translation coronal, pelvic tilt, hip pelvis lateral angulation, hip pelvis anterior translation coronal, and hip pelvis lateral translation (first row), and each of the physical performance skills (first column).

Physical Performance Skills	Rib Anterior Translation Coronal		Pelvic Tilt		Hip Pelvis Lateral Angulation		Hip Pelvis Anterior Translation Coronal		Hip Pelvis Lateral Translation	
	r	*p*	r	*p*	r	*p*	r	*p*	r	*p*
Stork balance test (s)	−0.41	<0.001	−0.35	0.02	−0.44	<0.001	−0.47	<0.001	−0.5	<0.001
Y-balance test (cm) anterior for the left side	−0.32	0.008	−0.36	0.025	−0.31	0.013	−0.45	<0.001	−0.46	<0.001
Y-balance test (cm) anterior for the right side	0.24	0.052	0.14	0.268	0.15	0.239	−0.062	0.623	0.03	0.796
Y-balance test (cm) posterolateral for the left side	−0.36	0.003	−0.43	<0.001	−0.39	0.001	−0.53	<0.001	−0.41	<0.001
Y-balance test (cm) posterolateral for the right side	−0.33	0.006	−0.42	<0.001	−0.39	0.001	−0.46	<0.001	−0.43	<0.001
Y-balance test (cm) posteromedial for the left side	−0.33	0.008	−0.42	<0.001	−0.36	0.002	−0.51	<0.001	−0.47	<0.001
Y-balance test (cm) posteromedial for the right side	−0.27	0.03	−0.32	0.009	−0.28	0.022	−0.42	<0.001	−0.39	0.001
Vertical jump test (cm)	−0.52	<0.001	−0.54	<0.001	−0.48	<0.001	−0.65	<0.001	−0.56	<0.001
Agility test (s)	0.75	<0.001	0.79	<0.001	0.79	<0.001	0.78	<0.001	0.73	<0.001

**Table 7:** Spearman correlation coefficient (r) and *p*-values (*p*) between CVA, lateral translation head sagittal, AHT coronal, lateral angulation head, and thoracic kyphosis (first row), and each of the cardiopulmonary exercise tests (first column).

Cardiopulmonary Functions	CVA		Lateral Translation Head Sagittal		AHT Coronal		Lateral Angulation Head		Thoracic Kyphosis	
	r	*p*	r	*p*	r	*p*	r	*p*	r	*p*
VE/VCO2 slope	−0.09	0.458	0.16	0.196	0.14	0.264	0.16	0.21	0.19	0.14
VO2 (mL/min) at VT	0.65	<0.001	−0.62	<0.001	−0.69	<0.001	−0.48	<0.001	−0.39	<0.001
VO2/kg	0.71	<0.001	−0.72	<0.001	−0.73	<0.001	−0.63	<0.001	−0.52	<0.001
VE I/min (VE/VO2 at VT)	−0.61	<0.001	0.65	<0.001	0.64	<0.001	0.591	<0.001	0.66	<0.001

**Table 8:** Spearman correlation coefficient (r) and *p*-values (*p*) between rib anterior translation coronal, pelvic tilt, hip pelvis lateral angulation, hip pelvis anterior translation coronal, and hip pelvis lateral translation (first row), and each of the cardiopulmonary exercise tests (first column).

Cardiopulmonary Functions	Rib Anterior Translation Coronal		Pelvic Tilt		Hip-Pelvis Lateral Angulation		Hip Pelvis Anterior Translation Coronal		Hip Pelvis Lateral Translation	
	r	*p*	r	*p*	r	*p*	r	*p*	r	*p*
VE/VCO2 slope	0.13	0.298	0.2	0.11	0.173	0.168	0.18	0.151	0.13	0.294
VO2 (mL/min) at VT	−0.52	<0.001	−0.62	<0.001	−0.58	<0.001	−0.63	<0.001	−0.6	<0.001
VO2/kg	−0.59	<0.001	−0.69	<0.001	−0.69	<0.001	−0.7	<0.001	−0.61	<0.001
VE I/min (VE/VO2 at VT)	0.65	<0.001	0.66	<0.001	0.66	<0.001	0.59	<0.001	0.59	<0.001

## Data Availability

The datasets analyzed in the current study are available from the corresponding author upon reasonable request.
